# Microbial Synergism Couples Root Metabolic Remodeling with Exudation Dynamics in *Liquidambar formosana*

**DOI:** 10.3390/microorganisms14061346

**Published:** 2026-06-16

**Authors:** Juan Song, Feng-Mao Chen, Li-Qin Zhang, Wei Gu, Cui-Yu Liu, Jia-Wei Liu, Yan-Qi Fan, Jia-Xin Ren, Xiang Wan, Jing-Han Wang

**Affiliations:** 1 School of Life Sciences, Huzhou Normal University, Huzhou 313000, China; 2 College of Forestry and Grassland, Nanjing Forestry University, Nanjing 210037, China; 3 Research Institute of Subtropical Forestry, Chinese Academy of Forestry, Hangzhou 311400, China

**Keywords:** *Liquidambar formosana*, *Funneliformis mosseae*, *Serratia marcescens*, metabolite profiling, root exudates

## Abstract

Plant–microbe interactions shape plant metabolism and rhizosphere processes, yet how root metabolic states are coupled to exudation remains unclear. Here, we show that inoculation with *Serratia marcescens* (NJ2D) and *Funneliformis mosseae* (BJ04), particularly in combination, enhances biomass (*p* < 0.005) and alleviates oxidative stress in *Liquidambar formosana*. Metabolomic analyses revealed a concerted remodeling of root primary metabolism, characterized by shifts in amino acids, organic acids, and sugars, alongside consistent enrichment of pentose and glucuronate interconversions. Concomitantly, root exudation was restructured, with increased release of carbon-rich metabolites. Notably, trehalose declined in both roots and exudates, indicating reduced osmoprotective demand and reallocation of metabolic resources. Together, these findings demonstrate that microbial inoculation reshapes root metabolism and exudation patterns in *L. formosana*, linking plant physiological responses with rhizosphere processes and potentially strengthening plant–microbe feedbacks.

## 1. Introduction

*Liquidambar formosana* Hance is a deciduous tree species widely distributed in temperate regions [1]. It possesses diverse medicinal properties and contains a range of bioactive compounds, particularly terpenoids, flavonoids, and phenolic acids, which are responsible for its pharmacological activities [2]. In addition, *L. formosana* has been identified as a promising natural pigment resource and has attracted increasing attention for its potential applications in the pharmaceutical and cosmetic industries [3]. Owing to its medicinal, economic, ecological, and ornamental value, *L. formosana* represents an important species with considerable prospects for sustainable utilization and industrial development. However, in recent decades, Asia has experienced significant climate change, characterized by increasing acid deposition [4], rising temperatures, and more frequent water-related stresses such as drought [5,6]. These environmental changes pose substantial challenges to the growth and survival of *L. formosana*. Furthermore, its productivity is constrained by additional factors, including soil nutrient deficiencies [7]. Given the growing demand for forest resources, ecological services, and bio-based products, there is an urgent need to better understand the adaptive responses and cultivation strategies of *L. formosana* under changing environmental conditions.

Interactions between plants and beneficial soil microorganisms play a central role in regulating plant metabolism, growth, and stress adaptation, with important implications for ecosystem functioning and sustainable agriculture [8,9,10]. Arbuscular mycorrhizal fungi (AMF) form intimate symbioses with plant roots, enhancing the acquisition of mineral nutrients and water in exchange for photosynthetically derived carbon, thereby improving plant growth and tolerance to abiotic and biotic stresses [11,12,13,14,15,16]. In parallel, plant growth-promoting rhizobacteria (PGPR), including *Serratia marcescens*, colonize the rhizosphere and phyllosphere and enhance plant performance through multiple mechanisms, such as phosphate solubilization, regulation of reactive oxygen species (ROS) homeostasis, siderophore production, and modulation of plant immune responses [17,18,19,20,21]. Despite substantial progress in understanding the individual contributions of AMF and PGPR to plant growth and stress responses, how these functionally distinct microbial partners interact to coordinately regulate plant metabolism remains poorly resolved. In particular, the extent to which AMF-PGPR co-inoculation drives metabolic reprogramming in host roots and alters root exudation patterns is largely unknown. Root exudates represent a primary interface between plants and the rhizosphere microbiome, functioning as both carbon sources and signaling molecules; changes in their composition can reshape microbial community assembly and establish feedbacks that influence plant physiological performance [22,23,24].

This study examines the combined effects of AMF and *S. marcescens* inoculation on *L. formosana*. By integrating phenotypic assessments with untargeted metabolomic profiling of root tissues and root exudates, we aim to elucidate the biochemical mechanisms underlying this tripartite interaction. Specifically, we seek to identify microbial-mediated metabolic pathways that enhance plant growth and stress resilience, thereby providing a mechanistic foundation for the sustainable cultivation and industrial utilization of this economically important tree species.

## 2. Materials and Methods

### 2.1. Microbial Strains

*Serratia marcescens* NJ2D, a plant growth-promoting bacterium, was isolated from the rhizosphere soil of 50-year-old *Liquidambar formosana* Hance in Nanjing, Jiangsu, China, and deposited in the China Center for Type Culture Collection (CCTCC; accession no. M 2012261). The arbuscular mycorrhizal fungus *Funneliformis mosseae* (BJ04) was provided by the Institute of Plant Nutrition and Resources, Beijing Academy of Agriculture and Forestry Sciences, China.

### 2.2. Plant Materials and Experimental Treatments

Surfaces of uniform, undamaged seeds of *L. formosana* were sterilized by immersion in 70% (*v*/*v*) ethanol for 2 min, followed by 3% (*w*/*v*) NaClO for 10 min, rinsed thoroughly with sterile distilled water, and then air-dried. Germinated seedlings were aseptically transferred to MS medium [25] solidified with agar in tissue culture vessels. Cultures were maintained in a growth chamber under a 16 h light/8 h dark photoperiod at 21 °C and 70% relative humidity.

Four treatments were established: (i) non-inoculated control (CK); (ii) inoculation with NJ2D alone (+NJ2D); (iii) inoculation with BJ04 alone (+BJ04); and (iv) co-inoculation with NJ2D and BJ04 (NJ2D_BJ04). Each treatment comprised 10 replicates, with a total of 40 tissue culture vessels arranged in a completely randomized design.

### 2.3. Plant Biomass and Root Colonization

One month after inoculation, *L. formosana* seedlings were harvested to evaluate plant biomass and AMF colonization. The seedlings were first weighed to determine their biomass. Subsequently, root samples were collected, and root segments approximately 1.0–1.5 cm in length were excised. Mycorrhizal colonization was assessed using the trypan blue staining method (0.05%, *w*/*v*) as previously described [26]. Three biological replicates were included for each treatment.

A green fluorescent protein (GFP)-tagged strain of *S. marcescens* was constructed to visualize and quantify bacterial colonization in *L. formosana* roots following established protocols [27,28]. The GFP plasmid was introduced into electrocompetent cells at the logarithmic growth phase via electroporation, and transformants were selected on LB plates supplemented with kanamycin (50 µg/mL) and verified by fluorescence microscopy (ZEISS LSM 900, Carl Zeiss Microscopy GmbH, Jena, Germany) (excitation 488 nm; emission 510–530 nm). The labeled strain was cultured to an OD600 = 0.6 and adjusted to 1 × 10^8^ CFU mL^−1^ for inoculation, and seedling roots were immersed in the suspension for 30 min. At 30 days post-inoculation, roots were gently washed three times with sterile phosphate-buffered saline (PBS) and examined using confocal laser scanning microscopy (ZEISS LSM 900, Carl Zeiss Microscopy GmbH, Jena, Germany; excitation 488 nm). Z-stack images were acquired for three-dimensional reconstruction to resolve bacterial distribution on the rhizoplane and within root tissues, and colonization density was quantified based on fluorescence intensity using ImageJ (version 1.53) with standardized thresholding. At least three biological replicates were included for each treatment.

### 2.4. Antioxidant Enzyme Activity Assays

Antioxidant enzyme activities were measured in *L. formosana* seedlings one month after microbial inoculation. Fresh seedlings (approximately 0.5 g) were rinsed, flash-frozen in liquid nitrogen, ground to a fine powder, and homogenized in 2.5 mL of cold 100 mM borate buffer (pH 8.8) containing 2% (*w*/*v*) polyvinylpyrrolidone (PVP). The homogenate was centrifuged at 11,304× *g* for 13 min at 4 °C, and the supernatant was used as the crude enzyme extract. Peroxidase (POD) activity was determined according to Yu et al. [29] by monitoring guaiacol oxidation in the presence of H_2_O_2_ at 470 nm, with one unit defined as the amount of enzyme causing an absorbance increase of 0.01 min^−1^ at 25 °C. Phenylalanine ammonia-lyase (PAL) activity was measured following Konappa et al. [30] by quantifying trans-cinnamic acid formation from L-phenylalanine at 290 nm. Superoxide dismutase (SOD) activity was assayed according to Lu et al. [31] based on the inhibition of nitroblue tetrazolium (NBT) photoreduction, with one unit defined as the amount of enzyme required to achieve 50% inhibition at 560 nm. Lipid peroxidation was estimated as malondialdehyde (MDA) content using the thiobarbituric acid (TBA) method described by Sun et al. [32], with absorbance measured at 532 nm and corrected at 600 nm. All enzyme activities and MDA content were expressed on a fresh weight basis, and measurements were performed with three biological replicates.

### 2.5. Sample Collection for Metabolomic Analysis

At 1 month post-inoculation, roots of *L. formosana* were excised into small segments, transferred into cryogenic tubes, immediately flash-frozen in liquid nitrogen, and transported to the laboratory. For each treatment, three independent biological replicates were harvested and analyzed. Samples were then stored at −80 °C until further analysis.

Approximately 100 mg of *L. formosana* root tissue was weighed into 1.5 mL polypropylene microtubes for sequential extraction of primary metabolites. Five steel beads were added, and samples were frozen in liquid nitrogen for 5 min, followed by homogenization at 70 Hz for 1 min using a high-throughput tissue grinder. Methanol (400 μL, pre-cooled to −20°C) was added, and samples were vortexed for 30 s. Ribitol (60 μL, 0.2 mg mL^−1^ in methanol) was then added as an internal standard, followed by vortexing for 30 s. Samples were sonicated at room temperature for 30 min, after which chloroform (750 μL, −20°C) and deionized water (1400 μL, 4 °C) were added and mixed thoroughly. After centrifugation (14,000 rpm, 4 °C, 10 min), 1 mL of the supernatant was transferred to a new tube and dried under vacuum. The residue was derivatized with methoxyamine in pyridine (60 μL, 15 mg mL^−1^) at 37°C for 120 min, followed by addition of BSTFA (60 μL, 1% TMCS) and incubation at 37°C for 90 min. After centrifugation (12,000 rpm, 4 °C, 10 min), the supernatant was transferred to GC vials for GC-MS analysis.

GC-MS analysis was performed using an HP-5MS capillary column (Agilent J&W Scientific, Folsom, CA, USA, 30 m × 250 μm i.d., 0.25 μm film thickness; 5% phenyl/95% methylpolysiloxane), with helium as the carrier gas at a constant flow rate of 1 mL min^−1^. Samples (1 μL) were injected in split mode (20:1). The injector, transfer line, and ion source temperatures were set at 280 °C, 150 °C, and 230 °C, respectively. The oven temperature was initially held at 60 °C for 2 min, ramped to 300 °C at 10 °C min^−1^, and maintained for 5 min. Mass spectra were acquired in full-scan mode over an m/z range of 35–750 [33,34].

### 2.6. Root Exudate Extraction and Analysis

Plantlets were cultured on MS agar medium as described above. After 1 month, whole *L. formosana* plantlets were removed from the medium and transferred to sterile distilled water for overnight incubation to collect root exudates. The exudate solution was subsequently freeze-dried and sent to Panomics (Bionovogene, Suzhou, China) for analysis.

Samples were thawed at 4 °C. An aliquot (100 μL) was transferred to a 1.5 mL microcentrifuge tube, mixed with 400 μL methanol, and vortexed for 60 s. Nonadecylic acid (60 μL, 0.2 mg mL^−1^ in methanol) and d4-alanine (60 μL, 10 mM in methanol) were added as internal standards, followed by vortexing for 60 s. Samples were centrifuged (12,000 rpm, 4 °C, 10 min), and the supernatant was transferred to a new tube and dried under vacuum. The residue was derivatized with methoxyamine in pyridine (60 μL, 15 mg mL^−1^) at 37 °C for 120 min, followed by addition of BSTFA (60 μL, 1% TMCS) and incubation at 37 °C for 90 min. After centrifugation (12,000 rpm, 4 °C, 10 min), the supernatant was transferred to GC vials for GC-MS analysis.

Samples were analyzed by GC–MS under the conditions described above [35,36].

### 2.7. Statistical Analyses

Data analysis was performed using SPSS 19.0 (IBM, Armonk, NY, USA). One-way ANOVA was used to evaluate differences in metabolite abundance among treatments in root tissues and rhizosphere exudates of *L. formosana*. Two-way ANOVA was conducted using NJ2D inoculation and BJ04 inoculation as fixed factors to assess their main and interaction effects on plant growth and physiological traits. Differential metabolites were identified using the combined thresholds of variable importance in projection (VIP) ≥ 1.0, fold change (FC) ≥ 1.5 or ≤0.667, and *p* < 0.05. Principal component analysis (PCA), heatmap visualization, and hierarchical clustering analyses were performed in R (software version 4.5.3) (R Core Team, Vienna, Austria), whereas box plots were generated using SigmaPlot version 15.0 (Systat Software Inc., San Jose, CA, USA).

## 3. Results

### 3.1. Plant Growth Parameters and Analysis of Mycorrhization

**Figure 1:**
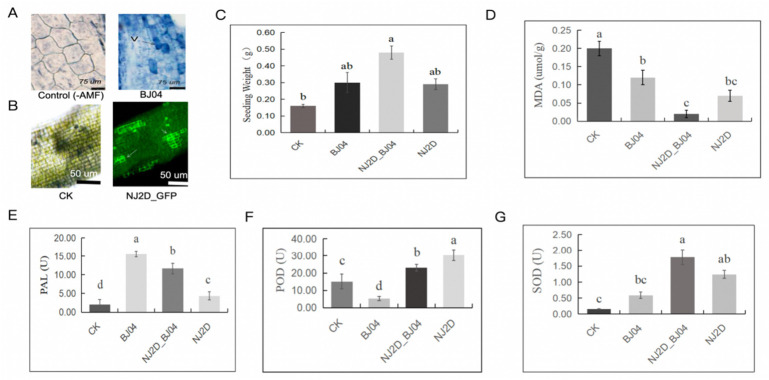
Colonization and growth-promoting effects of NJ2D and AMF on *L. formosana* seedlings. (**A**) AMF root colonization; (**B**) NJ2D root colonization (fluorescence imaging); (**C**) biomass accumulation; (**D**) MDA content; (**E**) PAL activity; (**F**) POD activity; (**G**) SOD activity. The arrow indicates the colonization site of GFP-labelled NJ2D in the root tissue. Different lowercase letters indicate significant differences among treatments at *p* < 0.05.

Arbuscular mycorrhizal fungi (AMF) successfully colonized the roots of *L. formosana* seedlings following BJ04 inoculation (Figure 1A). Consistently, fluorescence imaging confirmed effective root colonization by NJ2D, with strong green signals detected on the rhizoplane and within internal tissues, indicating stable bacterial establishment (Figure 1B). To assess plant growth-promoting effects, *L. formosana* seedlings were subjected to NJ2D, BJ04, and combined (NJ2D_BJ04) treatments. Notably, NJ2D_BJ04 significantly increased seedling biomass compared with the control (CK; *p* < 0.005) (Figure 1C). All inoculation treatments markedly reduced malondialdehyde (MDA) levels, with decreases of 74.00%, 35.00%, and 90.00% under NJ2D, BJ04, and NJ2D_BJ04, respectively, indicating alleviation of oxidative damage (Figure 1D). Correspondingly, antioxidant responses were enhanced, as phenylalanine ammonia-lyase (PAL) activity increased by 170.00% (NJ2D), 700.00% (BJ04), and 500.00% (NJ2D_BJ04) relative to the control (Figure 1E), while peroxidase (POD) activity rose by 146.15% (NJ2D) and 83.33% (NJ2D_BJ04) relative to the control (Figure 1F). Superoxide dismutase (SOD) activity was also significantly elevated in NJ2D and NJ2D_BJ04 treatments (*p* < 0.005) (Figure 1G). Significant interaction effects between NJ2D and BJ04 were detected only for PAL activity (*p* < 0.05), while no significant interaction effects were observed for seedling biomass, MDA, POD, or SOD (Table S1).

### 3.2. Metabolic Profiles in L. formosana Roots

**Figure 2:**
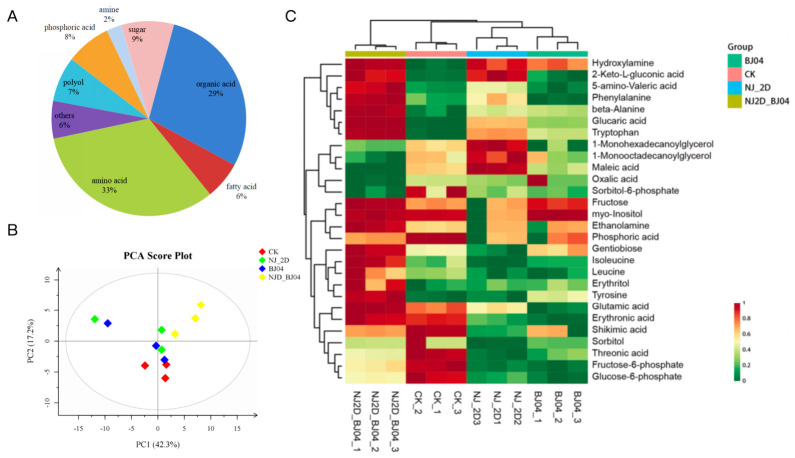
Microbial modulation of root metabolite profiles in *L. formosana*. (**A**) Metabolite classification; (**B**) PCA score plot; (**C**) heatmap of differential metabolites.

A total of 81 metabolites were identified by gas chromatography–mass spectrometry (GC-MS) and comprised predominantly primary metabolites, including amino acids (33%), organic acids (29%), sugars (9%), phosphoric acids (8%), polyols (7%), fatty acids (6%), and amines (2%). Amino acids, organic acids, sugars, and phosphoric acids together constituted the dominant metabolic classes (Figure 2A). Principal component analysis (PCA) revealed pronounced metabolic divergence among treatments, capturing both global variation and group-specific signatures. The PCA score plot (Figure 2B) showed clear and consistent separation among CK, NJ2D, BJ04, and NJ2D_BJ04 samples, with PC1 and PC2 explaining 42.3% and 17.2% of the total variance, respectively. Notably, the distinct clustering patterns indicate that microbial inoculation imposes a strong and differential metabolic imprint on root tissues. Collectively, these results suggest that NJ2D and BJ04, individually or in combination, drive a coordinated reconfiguration of root-derived primary metabolism, which may act as a key regulatory interface mediating downstream physiological and molecular responses in *L. formosana*.

**Figure 3:**
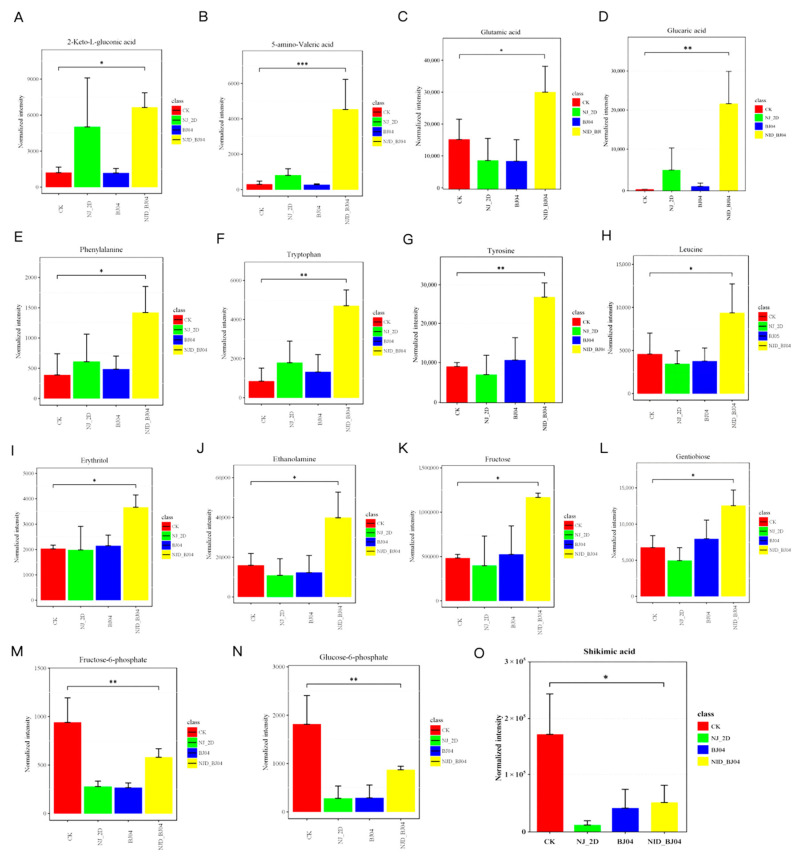
Significantly altered LC-MS metabolites in *L. formosana* roots following NJ2D and BJ04 inoculation. (**A**–**O**) Relative abundance of key metabolites, including 2-keto-L-gluconic acid, 5-aminovaleric acid, glutamic acid, glucaric acid, phenylalanine, tryptophan, tyrosine, leucine, erythritol, ethanolamine, fructose, gentiobiose, fructose-6-phosphate, glucose-6-phosphate, and shikimic acid. Significance levels are indicated as *** *p* < 0.001, ** *p* < 0.01, * *p* < 0.05.

Heatmap clustering further highlighted pronounced treatment-specific metabolic reprogramming (Figure 2C). Relative to the CK, NJ2D inoculation led to a marked reduction in several metabolites, including shikimic acid, threonic acid, fructose-6-phosphate, erythronic acid, sorbitol, and glucose-6-phosphate. In contrast, NJ2D significantly increased the accumulation of glucaric acid, 2-keto-L-gluconic acid, hydroxylamine, 1-monohexadecanoylglycerol, 1-monooctadecanoylglycerol, maleic acid, phenylalanine, and tryptophan (Figure 2C). BJ04 treatment exhibited a distinct metabolic signature, characterized by elevated levels of hydroxylamine, tryptophan, fructose, tyrosine, and β-alanine compared with the CK. Notably, the combined NJ2D_BJ04 treatment induced a broader and more pronounced metabolic shift, with significant increases in 5-aminovaleric acid, glucaric acid, tryptophan, tyrosine, β-alanine, gentiobiose, fructose, glutamic acid, isoleucine, ethanolamine, erythritol, 2-keto-L-gluconic acid, and phenylalanine (*p* < 0.05; Figure 3). Conversely, shikimic acid and glucose-6-phosphate were consistently reduced in the NJ2D_BJ04 treatment relative to the CK (*p* < 0.05; Figure 3).

### 3.3. Differentially Abundant Metabolite KEGG Pathways

**Figure 4:**
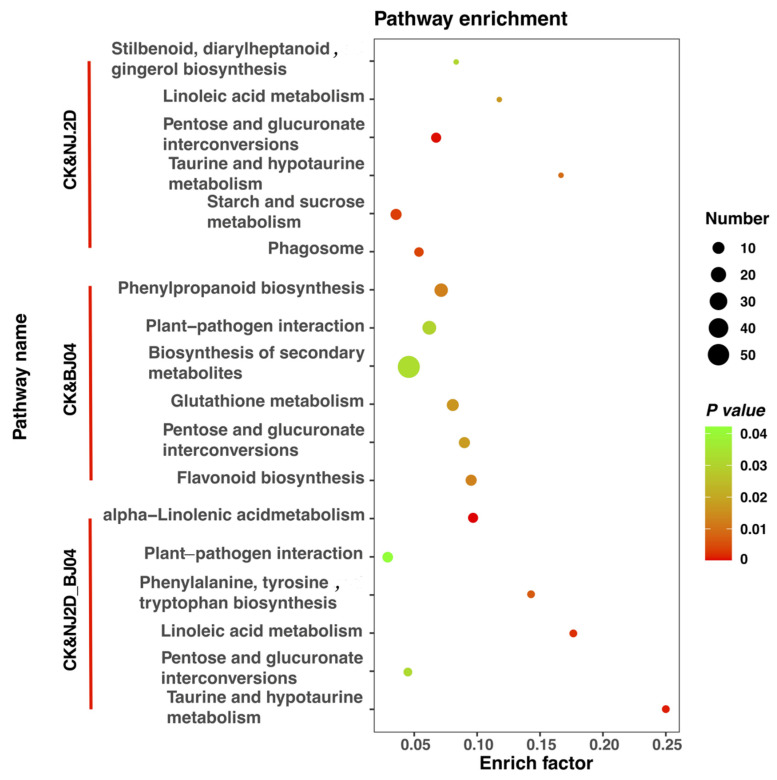
KEGG pathway enrichment reveals microbiome-driven metabolic reprogramming in *L. formosana* roots. Dot size indicates the number of enriched metabolites and color denotes significance (redder = lower *p* values; *p* < 0.05). Comparisons include CK vs. NJ2D, BJ04, and NJ2D_BJ04.

All metabolites were significantly impacted by NJ2D, BJ04, and NJ2D_BJ04 treatments, which were mapped to biological pathways using the KEGG database. The most differentiated metabolites were selected for comparative analysis (Figure 4). In the CK vs. NJ2D group, key pathways included stilbenoid, diarylheptanoid, and gingerol biosynthesis; linoleic acid metabolism; pentose and glucuronate interconversions; taurine and hypotaurine metabolism; starch and sucrose metabolism; and phagosome. In the CK vs. BJ04 group, pathways included phenylpropanoid biosynthesis; plant–pathogen interaction; biosynthesis of secondary metabolites; glutathione metabolism; pentose and glucuronate interconversions; and flavonoid biosynthesis. In the CK vs. NJ2D_BJ04 group, the most notable pathways were alpha-linolenic acid metabolism; plant–pathogen interaction; phenylalanine, tyrosine, and tryptophan biosynthesis; linoleic acid metabolism; pentose and glucuronate interconversions; and taurine and hypotaurine metabolism (Figure 4). Notably, the pentose and glucuronate interconversions pathway was common across all comparisons.

### 3.4. Analysis of Root Exudate Components in L. formosana

**Figure 5:**
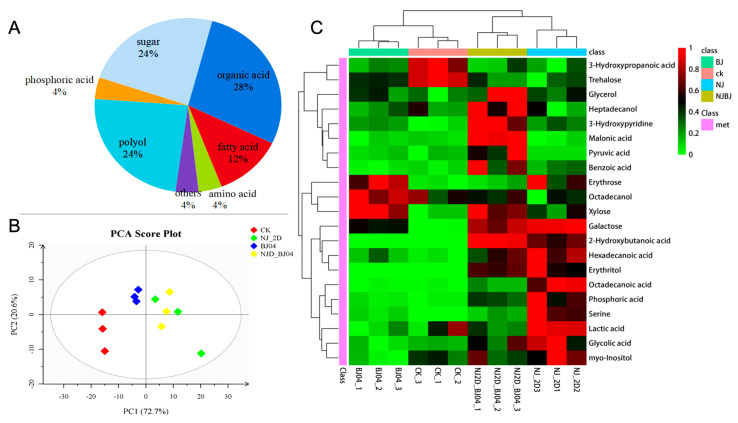
Microbial synergy reshapes root exudate chemistry in *L. formosana*. (**A**) Classification of root exudates; (**B**) PCA of exudate profiles; (**C**) heatmap of differential metabolites.

**Figure 6:**
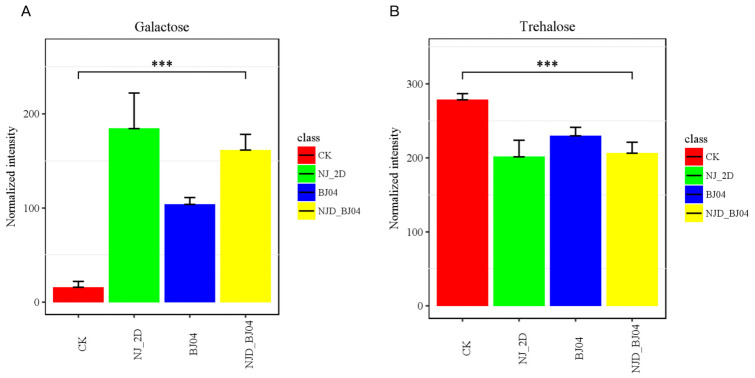
Microbial regulation of carbohydrate allocation in *L. formosana* root exudates. (**A**) Galactose; (**B**) trehalose. Significance levels are indicated as *** *p* < 0.001.

The root exudates of *L. formosana* were predominantly composed of organic acids (28%), sugars (24%), and polyols (24%), followed by fatty acids (12%), amino acids (4%), phosphoric acid (4%), and other minor compounds (Figure 5A), indicating a carbon-rich exudation profile. Principal component analysis (PCA) revealed a clear separation among treatments, with PC1 and PC2 explaining 72.7% and 20.6% of the total variance, respectively (Figure 5B), suggesting that microbial inoculation substantially reshaped the root exudate composition. Consistently, heatmap clustering highlighted pronounced treatment-specific shifts in metabolite profiles (Figure 5C). NJ2D inoculation markedly increased the accumulation of carbohydrates (e.g., galactose), organic acids (e.g., 2-hydroxybutanoic acid, lactic acid, glycolic acid), fatty acids (e.g., hexadecanoic acid, octadecanoic acid), and polyols (e.g., erythritol, myo-inositol), suggesting enhanced carbon allocation and metabolic activity in the rhizosphere. BJ04 treatment preferentially enriched sugars and sugar alcohol-related metabolites, including erythrose, galactose, and xylose, indicating a shift toward carbohydrate-dominated exudation patterns. Notably, co-inoculation (NJ2D_BJ04) induced a broader and more pronounced metabolic reprogramming, characterized by significant increases in heptadecanol, 3-hydroxypyridine, malonic acid, xylose, galactose, 2-hydroxybutanoic acid, and erythritol (*p* < 0.05; Figure 5C and Figure 6). These results collectively indicate that the microbial consortium may exert a synergistic effect in modulating primary metabolism. Furthermore, trehalose accumulation was significantly reduced in all inoculated treatments compared with the CK (*p* < 0.05; Figure 6), suggesting a reduced requirement for osmoprotective metabolites. This decline may reflect an improved physiological status of the host or microbial modulation of primary metabolism, which may be associated with altered stress adaptation and microbe–host signaling processes. Overall, these results demonstrate that microbial inoculation, particularly co-inoculation, drives a coordinated reconfiguration of root exudate composition, likely contributing to rhizosphere metabolic interactions and plant–microbe feedback regulation.

### 3.5. Functional Pathway Analysis of Differentially Abundant Root Exudates

The KEGG database was used to map differentially accumulated metabolites (DAMs) from the NJ2D vs. CK, BJ04 vs. CK, and NJ2D_BJ04 vs. CK comparisons of metabolic pathways. Although only a small number of DAMs were assigned to individual pathways, several functionally relevant categories were identified. Notably, starch and sucrose metabolism (ko00500) was the only pathway shared across all treatments (Table 1). Given the limited number of mapped metabolites, these results should be interpreted as indicative of targeted metabolic adjustments rather than robust pathway-level enrichment. Nevertheless, the recurrent involvement of carbohydrate metabolism suggests a potential role in mediating treatment-induced changes in root exudation.

### 3.6. Linking Root Metabolic Shifts to Rhizosphere Exudation

**Figure 7:**
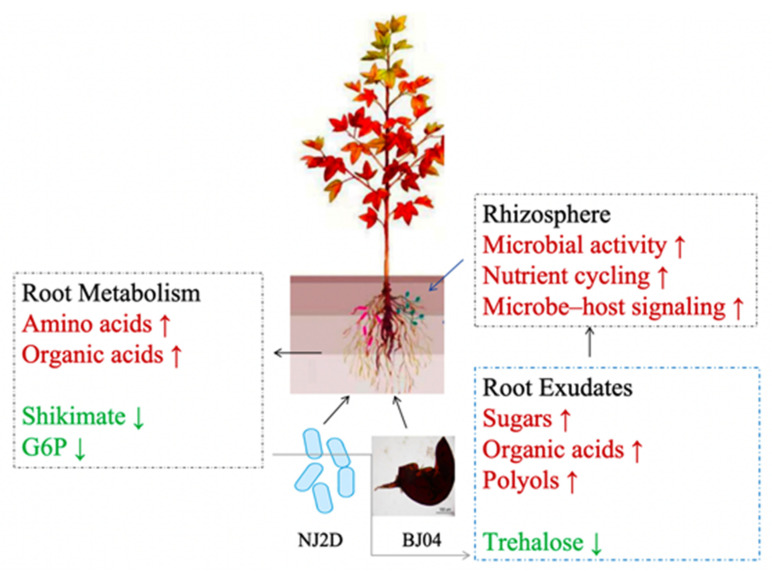
Conceptual model illustrating microbial consortium-driven reprogramming of root metabolism and exudation in *L. formosana*. Red and green arrows indicate changes in abundance: red arrows represent increase (↑), and green arrows represent decrease (↓).

Root tissues were characterized by a high proportion of amino acids (33%) and organic acids (29%), while root exudates displayed a carbon-enriched profile comprising organic acids (28%), sugars (24%), and polyols (24%) (Figure 2A and Figure 5A). Principal component analysis indicated that microbial treatments induced stronger metabolic shifts in exudates (93.3% variance explained) compared to tissues (59.5%) (Figure 2B and Figure 5B). Coordinated accumulations of tryptophan, phenylalanine, erythritol, and 2-keto-L-gluconic acid were detected in both compartments under inoculation. In contrast, shikimic acid and glucose-6-phosphate were reduced only in tissues, while trehalose declined exclusively in exudates (Figure 7), suggesting independent regulatory mechanisms. Together, these results reveal a coordinated albeit compartment-specific metabolic landscape shaped by microbial inoculation.

## 4. Discussion

Arbuscular mycorrhizal fungi (AMF) and plant growth-promoting rhizobacteria (PGPR) are key components of the soil microbiota, and both single and combined inoculations have been widely reported to enhance plant growth [37,38,39,40]. Consistent with these findings, our results showed that all inoculation treatments significantly increased the aboveground biomass of *L. formosana* seedlings compared with the control. Notably, co-inoculation resulted in greater increases than single inoculation, with the NJ2D_BJ04 treatment exhibiting the highest values across most measured indices. This pattern indicates a synergistic rather than additive interaction between NJ2D and BJ04, potentially driven by functional complementarity. AMF is known to enhance nutrient acquisition, particularly phosphorus, whereas PGPR can stimulate root development and nutrient mobilization. Previous studies have identified *Pseudomonas* spp. and *Bacillus* spp. as bacteria that facilitate AMF colonization and function [39,41]. In line with this, NJ2D may facilitate AMF colonization and function, thereby enhancing BJ04-mediated growth promotion, although further studies are required to confirm the underlying mechanisms.

Reactive oxygen species (ROS) act as central regulators of plant metabolism and development, with their homeostasis tightly controlled to balance signaling and oxidative damage [42]. Plants mitigate excessive ROS through an antioxidant defense system involving enzymes such as POD, PAL, and SOD, while malondialdehyde (MDA) reflects the extent of membrane lipid peroxidation [43]. Increasing evidence indicates that AMF can modulate this redox balance by enhancing antioxidant capacity, thereby reducing ROS accumulation while stabilizing cellular processes [44,45,46,47]. Consistent with this, our results showed that NJ2D, BJ04, and NJ2D_BJ04 treatments significantly decreased MDA content and increased POD, PAL, and SOD activities in *L. formosana* roots. These coordinated changes suggest an improved redox homeostasis, which may facilitate metabolic reprogramming toward more efficient resource allocation and cellular functioning. Notably, the stronger responses under co-inoculation indicate a synergistic effect, potentially amplifying redox-mediated metabolic adjustments and thereby contributing to enhanced plant growth.

Following the observed increases in biomass and antioxidant enzyme activities, metabolomic profiling was conducted to elucidate the mechanisms underlying growth promotion. Microbial inoculation significantly altered the metabolic composition of *L. formosana*, with multiple differential metabolites (*p* < 0.05) showing positive associations with plant growth (Figure 3). Among these, amino acids were prominently enriched, suggesting a central role in mediating plant responses. Amino acids are essential for protein synthesis, serve as precursors for phytohormones, and contribute to stress resistance, thereby supporting plant growth and development [48,49]. Consistently, amino-acid-based biostimulants have been shown to enhance crop yield and quality [50]. In this study, key amino acids, such as tryptophan and tyrosine, may promote cell division and elongation through their roles in hormone biosynthesis and metabolic regulation, while also acting as precursors of secondary metabolites involved in defense and signaling [51,52]. Additionally, phenylalanine is linked to lignin biosynthesis and anthocyanin production [53].

It should be noted that the collection of root exudates via overnight incubation in sterile distilled water may induce physiological stress, potentially altering the composition and abundance of released metabolites. Therefore, while the observed metabolomic changes provide valuable insights into microbial-mediated growth promotion, the exudate profiles may not fully reflect in situ conditions. Collectively, these results suggest that NJ2D and BJ04 facilitate growth by modulating amino acid metabolism, thereby enhancing metabolic capacity and developmental processes in *L. formosana*.

Root exudation plays a central role in mediating plant–environment interactions [54]. These exudates comprise diverse compounds, including sugars, organic acids, amino acids, and phenolics, which function in nutrient exchange and chemical signaling [55]. Through such signaling processes, plants and plant growth-promoting microorganisms (PGPM) establish dynamic interactions that influence mutual responses and rhizosphere functioning [56]. In this study, galactose levels were significantly elevated under NJ2D, BJ04, and NJ2D_BJ04 inoculation compared with the CK. Beyond its role as a carbon source, galactose contributes to cell wall biosynthesis and the formation of storage oligosaccharides, thereby supporting plant growth and stress tolerance [57,58]. Moreover, galactose has been implicated in mediating plant–microbe interactions by promoting bacterial colonization, as reported for *Bacillus* velezensis SQR9 [59]. Consistent with this, our findings suggest that increased galactose exudation may facilitate the establishment of NJ2D and BJ04, thereby reinforcing plant–microbe interactions and contributing to enhanced growth performance in *L. formosana*.

## 5. Conclusions

Co-inoculation with *Serratia marcescens* NJ2D and *Funneliformis mosseae* BJ04 maximized *Liquidambar formosana* growth via coordinated regulation of redox homeostasis, primary metabolism, and root exudation. Enhanced antioxidant capacity mitigated oxidative stress, while systemic reprogramming of amino acid, organic acid, and carbohydrate metabolism supported biomass accumulation. Parallel shifts in exudate composition revealed tight coupling between root metabolic state and rhizosphere processes. These findings demonstrate that microbial synergism functionally links root metabolic reprogramming to exudation dynamics, providing a mechanistic basis for plant growth promotion and highlighting the potential of synthetic microbial consortia as sustainable enhancers of tree productivity and ecosystem resilience.

## Figures and Tables

**Table 1:** KEGG pathway mapping of differentially accumulated root exudate metabolites.

Pathway Name	KEGG ID	NJ2D vs. CK (Hits)	BJ04 vs. CK (Hits)	NJ2D_BJ04 vs. CK (Hits)	Total Metabolites	Impact	Adjusted *p* Value (FDR)	Shared
Starch and sucrose metabolism	ko00500	1	1	2	30	0.09079	0.13499	Yes
Galactose metabolism	ko00052	0	0	1	26	0.01631	1	No

Notes: “Hits” indicate the number of differentially abundant metabolites mapped to each pathway; “Total metabolites” represent the total number of annotated metabolites in the corresponding KEGG pathway; and “0” indicates no significantly enriched metabolites detected in that comparison.

## Data Availability

The original contributions presented in this study are included in the article. Further inquiries can be directed to the corresponding author.

## Supplementary Materials

The following supporting information can be downloaded at: https://www.mdpi.com/article/10.3390/microorganisms14061346/s1, Table S1. Two-way ANOVA examining the effects of NJ2D, BJ04 and their interaction on physiological traits of *L. formosana*.
