# Supplementary material for: Microbial Synergism Couples Root Metabolic Remodeling with Exudation Dynamics in Liquidambar formosana
Source: Microorganisms. 2026 Jun 16;14(6):1346. doi: 10.3390/microorganisms14061346 (PMC13305431; doi:10.3390/microorganisms14061346)
Supplement: Supplementary file 1 [file microorganisms-14-01346-s001.zip › microorganisms-4344408-supplementary.pdf]

Table S1. Two-way ANOVA examining the effects of NJ2D, BJ04 and their interaction on physiological traits of *L. formosana.*

Trait	NJ2D (p)	BJ04 (p)	NJ2D × BJ04 (p)
Seedling weight	0.056	0.118	0.801
PAL	0.494	<0.001	0.022*
MDA	0.050	0.234	0.793
POD	<0.001	0.012	0.307
SOD	0.064	0.382	0.927

Note*:* indicates significant interaction at $p < 0.05$.
